# Supplementary material for: Glycation of Tie-2 Inhibits Angiopoietin-1 Signaling Activation and Angiopoietin-1-Induced Angiogenesis
Source: Int J Mol Sci. 2022 Jun 27;23(13):7137. doi: 10.3390/ijms23137137 (PMC9266685; doi:10.3390/ijms23137137)
Supplement: Supplementary file 1 [file ijms-23-07137-s001.zip › ijms-1771162-supplementary.pdf]

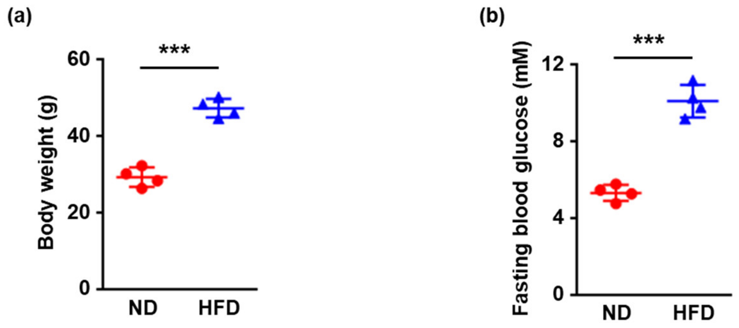

Figure S1. HFD induced obesity and hyperglycemia. 8 weeks old male C57BL/6J mice were fed with a HFD or a ND diet for 16 weeks. The body weight (**a**) and the fasting blood glucose (**b**) were detected before the aortae were separated. ***p < 0.01. Error bar represents the standard deviation and p value was generated by t test.

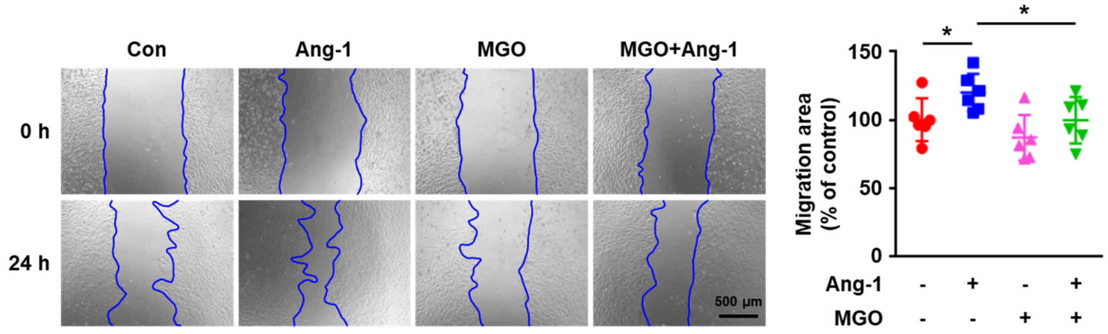

Figure S2. MGO inhibits Ang-1-induced endothelial cell migration. HUVECs were seeded onto 24-well plates, allowed to grown to 90% confluence and exposed to MGO (100 μM) for 24 h. Then the cells were scratched with a 200 μL pipette tip, followed by stimulation with Ang-1 (200 ng/mL) for 24 h. Photomicrographs were taken immediately after the scratch and after Ang-1 treatment. Representative images of 6 independent experiments are shown. The cell migration area was calculated and quantitative assessment was performed. $^*p < 0.05$. Error bar represents the standard deviation and p value was generated by One-way ANOVA followed by post hoc comparison.
